# Therapeutic Value of Voltage-Gated Sodium Channel Inhibitors in Breast, Colorectal, and Prostate Cancer: A Systematic Review

**DOI:** 10.3389/fphar.2015.00273

**Published:** 2015-11-12

**Authors:** Fabiola Martin, Chiedu Ufodiama, Ian Watt, Martin Bland, William J. Brackenbury

**Affiliations:** ^1^Department of Biology, University of YorkYork, UK; ^2^Hull York Medical School, University of YorkYork, UK; ^3^Department of Health Sciences, University of YorkYork, UK

**Keywords:** anticonvulsants, breast neoplasms, colonic neoplasms, prostatic neoplasms, sodium channels

## Abstract

Although survival rates of breast, colon, and prostate cancers are improving, deaths from these tumors frequently occur due to metastasis. Voltage-gated Na^+^ channels (VGSCs) are membrane proteins, which regulate membrane current and cellular migration during nervous system organogenesis. VGSCs are also expressed in fibroblasts, immune cells, glia, and metastatic cancer cells. VGSCs regulate migration and invasion of breast, bowel, and prostate cancer cells, suggesting that they may be novel anti-metastatic targets. We conducted a systematic review of clinical and preclinical studies testing the effects of VGSC-inhibiting drugs in cancer. Two-hundred and four publications were identified, of which two human, two mouse, and 20 *in vitro* publications were included. In the clinical studies, the effect of these drugs on survival and metastatic relapse is not clear. The 22 preclinical studies collectively suggest that several VGSC-inhibiting drugs inhibit cancer proliferation, migration, and invasion. None of the human and only six of the preclinical studies directly investigated the effect of the drugs on VGSC activity. Studies were difficult to compare due to lack of standardized methodology and outcome measures. We conclude that the benefits of VGSC inhibitors require further investigation. Standardization of future studies and outcome measures should enable meaningful study comparisons.

## Introduction

Cancers of the breast, colon, and prostate collectively account for the majority of cancer diagnoses in adults in the Western world (Jemal et al., 2011). Although survival rates are improving, deaths from these cancers frequently occur due to metastasis. Metastasis is a complex process, which involves detachment of cancer cells from the primary site, local invasion into surrounding tissues and dissemination to distant sites in other tissues. Metastatic disease is invariably incurable, and the molecular mechanisms underlying metastasis are not yet fully understood. Thus, there is an urgent need to develop new molecularly targeted anti-metastatic therapies with curative intent. Voltage-gated Na^+^ channels (VGSCs) are heteromeric membrane protein complexes made up of a single pore-forming α subunit (Na_v_1.1–Na_v_1.9) and one or more smaller auxiliary β subunits (β1–β4; Catterall, 2000). The β subunits contain an extracellular immunoglobulin loop and do not form part of the ion-conducting pore. Instead, they modulate channel gating and function as cell adhesion molecules (Brackenbury and Isom, 2011). The influx of Na^+^ ions through VGSCs is responsible for the membrane depolarization phase underlying action potentials in electrically excitable neurons and muscle cells. VGSC α and β subunits have also been shown to regulate several key aspects of organogenesis in the developing central nervous system, including cell proliferation, neurite outgrowth, neuronal pathfinding, and migration (Brackenbury et al., 2008a, 2010, 2013). Abnormal function of VGSCs contributes to various excitability-related pathologies, including epilepsy, cardiac arrhythmia, and neuropathic pain. As a result, a number of drugs have been developed to target VGSCs (Mantegazza et al., 2010).

Voltage-gated Na^+^ channels are also expressed in cells that are traditionally considered to be “non-excitable”, including fibroblasts, immune cells, glia, and metastatic cancer cells (Black and Waxman, 2013). In metastatic cancer cells, including those of the breast, bowel, and prostate, VGSCs have been shown to potentiate a number of cellular behaviors associated with metastasis, including migration and invasion (Brackenbury et al., 2008b; Brackenbury, 2012; Besson et al., 2015). Furthermore, emerging preclinical data suggest that pharmacologically targeting VGSCs may reduce local invasion and metastasis in mouse models (Driffort et al., 2014; Nelson et al., 2015). The potential utility of VGSC-inhibiting agents as anti-metastatic therapies has not surfaced in the clinic. However, the preclinical data raise the intriguing possibility that cancer patients taking VGSC-inhibiting medication for other pre-existing indications, e.g., epilepsy, may have improved cancer-specific outcome compared with those not taking such medications (Fairhurst et al., 2014).

Although a number of recent reviews have explored the literature relating to the contribution of VGSCs to metastasis (Roger et al., 2006; Brackenbury and Isom, 2008; Brackenbury et al., 2008b; Brackenbury, 2012; Fraser et al., 2014a; Besson et al., 2015), there has been no systematic review assessing the evidence for the potential therapeutic use of VGSC-inhibiting agents in cancer. We therefore set out to conduct a systematic review of the current clinical and preclinical studies that have been performed using known VGSC-inhibiting drugs in cancer cells. We have focused the review on cancers of the breast, bowel, and prostate because VGSC expression has been most extensively characterized in these tumors (Brackenbury, 2012). We have identified two clinical studies that explored the effect of VGSC inhibitors on clinical characteristics in cancer patients. However, the effect of these drugs on survival and metastatic relapse is not clear. Nonetheless, our search uncovered 22 preclinical studies collectively suggesting that several VGSC-inhibiting drugs inhibit various aspects of the hallmarks of cancer, including proliferation, angiogenesis, and invasion.

## Materials and Methods

### Search Strategy

A systematic literature search was performed to identify studies using VGSC-inhibiting drugs as part of the treatment of patients with colorectal, breast, and prostate cancer. The databases searched were Medline and Embase (Ovid interface) from inception until May 20th, 2015. Controlled vocabulary and free text terms were used in these search strategies. The search terms used were “[Sodium Channel blocking drugs] AND [colorectal cancer or breast cancer or prostate cancer]”. VGSC-inhibiting drugs included in the search are in **Table 1**. No limits or methodological filters were applied to these searches in order to avoid bias. The full search strategies are listed in Supplementary Tables S1 and S2. The protocol for the search strategy was registered with PROSPERO (registration number CRD42014013574).

**Table 1:** Drug search terms used in systematic review.

Carbamazepine
Carbamazepine derivatives and other carboxamides
Class Ib anti-arrhythmic agents
Disopyramide
Eslicarbazepine acetate
Flecainide
Lacosamide
Lamotrigine
Lidocaine
Mexiletine
Moricizine
Oxcarbazepine
Phenytoin
Procainamide
Propafenone
Quinidine
Ranolazine
Riluzole
Rufinamide
Sodium channel blocking drugs
Sodium valproate
Tocainide
Topiramate
Valproic acid

### Selection

**FIGURE 1:**
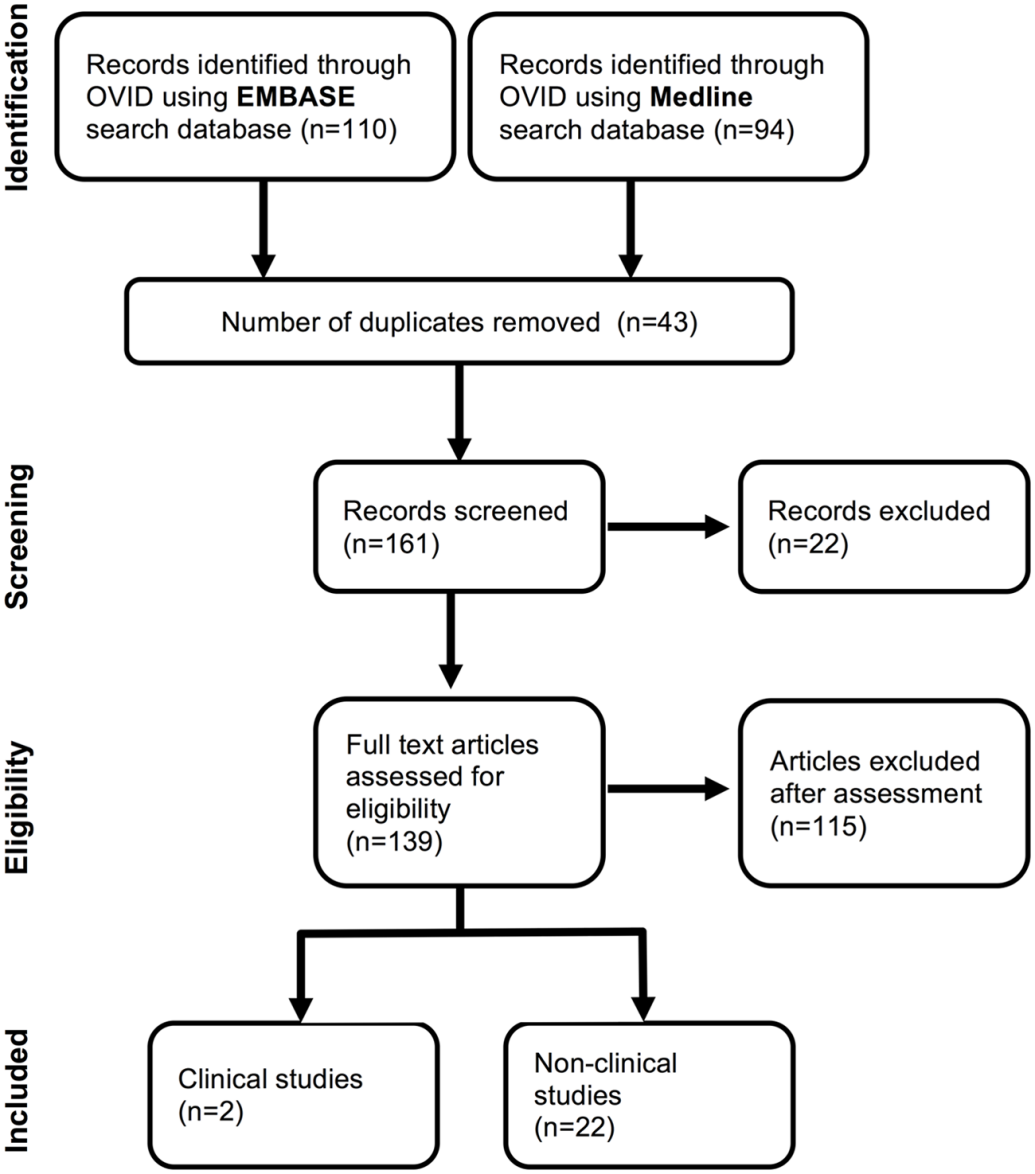
**Flow chart illustrating selection of studies for systematic review**. Initial exclusions were made following assessment of abstracts. Further studies were excluded after full text assessment due to language restrictions or study not looking at the effects of a voltage-gated Na^+^ channels (VGSC)-inhibiting drug listed in **Table 1**. The final shortlist of studies was achieved by scoring study quality and evidence against the standardized pre-piloted criteria in Supplementary Table S3. A minimum score of 3/5 was required for inclusion.

The PRISMA guidelines were used as a basis for the selection (Moher et al., 2009). Bibliographic details and their respective abstracts were downloaded into EndNote. Studies were then selected through a four-step process (**Figure 1**). The initial step was the identification of the studies from EMBASE and MEDLINE. This was achieved by reviewing the title of each study. The second step of the process involved identifying and removing any duplicates, removing of conference abstracts and obvious false selections. The third step selected manuscripts by the following inclusion criteria: the VGSC-inhibiting drug that was used, cancer was of the breast and/or colorectum and/or prostate, participants were over the age of 18 years. The exclusion criteria were: non-English abstract, study not peer-reviewed. At this stage, the full text was reviewed.

In the fourth step of the selection process, we modified a standardized Quality Assessment Tool for Observational Cohort and Cross-Sectional Studies (mQATSO) to quantify study quality and evidence (Supplementary Table S3; NHLBI, 2014). The following criteria each received a score of one: human studies = 1, using at least one VGSC-inhibitor = 1, at least one of the index cancers = 1, measuring cancer survival, and/or metastasis outcome = 1, specifically investigating the VGSC-inhibiting mode of action of the drug(s) = 1. Thus, a maximum score of 5 could be achieved. All data were collected in a spreadsheet in Microsoft Excel. Three reviewers (FM, CU, and WB) screened studies independently and then discussed and resolved discrepancies together.

## Results

Using search terms detailed in the section “Materials and Methods”, 204 items were identified in EMBASE and Medline, 43 of which were duplicates and were removed. Abstract screening excluded 22 additional records for not meeting inclusion criteria. Full manuscript screening contained 139 items, and 115 were excluded scoring <3/5 on quality assessment (**Figure 1**). Two human and 22 preclinical *in vivo* and *in vitro* publications were included in the final study.

The two human studies investigated a VGSC inhibitor in one of the cancers of interest and tested drug influence on cancer survival. However, neither of the studies tested specifically the VGSC-inhibiting activity of the intervention and therefore scored 4/5. Raderer et al. (1993) conducted an observational study of quinidine as a multi-drug resistance modifier adjuvant to pirarubicin in 14 women with metastatic and/or refractory breast cancer to test side-effects and survival outcomes, but an objective survival benefit was not observed. Wheler et al. (2014) conducted a phase 1 dose finding study of sodium valproate as adjunctive therapy to bevacizumab in 57 patients with cancer, 40 of whom had colon, breast, or prostate cancer. They attributed the survival benefits detected with sodium valproate to its histone deacetylase inhibition activity, which was dose independent (**Table 2**), (Wheler et al., 2014).

**Table 2:** Summary of included studies.

Reference	Population	Study design	Intervention	Outcome	Score /5
Raderer et al., 1993	Fourteen females with advanced refractory breast cancer	Phase I/II clinical trial of quinidine and pirarubicin	Two-hundred and fifty milligram quinidine bisulphate twice daily for 5 days. Cycle repeated every 3–4 weeks.	Stable disease in six patients, progression in eight patients	4
Wheler et al., 2014	Fifty-seven patients with colorectal (51%), prostate (10%), breast (9%), or other cancer (31%)	Phase I trial of bevacizumab and VPA in patients with untreatable advanced cancer	Valproic acid 5.3 mg/kg once daily + bevacizumab 11 mg/kg every 14 days	Safe combination. Improved overall survival if hypertension present	4
Abdul and Hoosein, 2001	LNCaP, PC-3 and DU-145 prostate cancer cell lines	*In vitro* study of drug effect on proliferation	Phenytoin, carbamazepine, valproate	Drugs inhibited proliferation at clinically relevant doses	4
Abdul and Hoosein, 2002	LNCaP, PC-3, DU-145, and MDA-PCA-2B prostate cancer cell lines	*In vitro* study of drug effect on proliferation	Riluzole	Riluzole inhibited proliferation	4
Anderson et al., 2003	PC-3	Compound discovery of phenytoin analogs	Phenytoin and analogs	Phenytoin and synthesized analogs inhibit proliferation	4
Driffort et al., 2014	Spontaneous metastasis murine model using MDA-MB-231 breast cancer cells	*In vivo* study of drug effect on lung metastasis	Ranolazine	Ranolazine inhibits lung metastasis *in vivo* and Na^+^ current, invasion and extracellular matrix degradation *in vitro*	4
Nelson et al., 2015	Orthotopic murine breast cancer model using MDA-MB-231 breast cancer cells	*In vivo* study of drug effect on breast tumor growth, invasion, and metastasis	Phenytoin 60 mg/kg once daily	At clinically relevant dose, phenytoin reduces tumor growth, proliferation, invasion, and metastasis	4
Yang et al., 2012	MCF-7 and MDA-MB-231 breast cancer cells	*In vitro* study of drug effect on Na^+^ current, migration, and invasion	Phenytoin	Phenytoin inhibits migration and invasion of VGSC-expressing MDA-MB-231 cells	4
Al Snafi et al., 2014	AMN-3 breast cancer cells	*In vitro* study of drug effect on cell viability	Valproate	Valproate inhibits cell proliferation	3
Angelucci et al., 2006	LNCaP, DU145, PC-3 prostate cancer cells	*In vitro* study of drug effect on growth and apoptosis	Valproic acid and butyrate analogs	Valproic acid inhibits cell growth and stimulates programmed cell death	3
Chang et al., 2014	MCF-7 mammary carcinoma and MCF-10A epithelial cells	*In vitro* study of drug effect on apoptosis	Lidocaine, tetracaine	Drugs inhibited morphological changes but were not pro-apoptotic	3
Fortunati et al., 2008	MCF-7, ZR-75-1, MDA-MB-231, and MDA-MB-435 breast cancer cells	*In vitro* study of drug effect on proliferation	Valproic acid	Valproic acid inhibited proliferation in estrogen-sensitive breast cancer cells	3
Iacopino et al., 2008	LNCaP; PC-3 prostate cancer cells	*In vitro* study of drug effect on proliferation	Valproic acid	Valproic acid inhibited proliferation in both cell lines to a variable extent	3
Jafary et al., 2014	MCF-7 breast cancer cells	*In vitro* study of drug effect on proliferation	Valproic acid + nicotinamide	Drug combination inhibited proliferation, increased apoptosis	3
Jawed et al., 2007	MCF-7 breast cancer cells	*In vitro* study of drug effect on proliferation	Valproic acid + melatonin	Valproic acid inhibited proliferation in presence/absence of melatonin	3
Jiang et al., 2014	PC3, DU145 prostate cancer cells	*In vitro* study of drug effect on invasion and SMAD4 activity	Valproic acid	Valproic acid inhibited invasion through AKT pathway	3
Li et al., 2012	MDA-MB-231 breast cancer cells	*In vitro* study of drug effect on cell behaviors	Valproic acid	Valproic acid inhibited cell migration but not proliferation	3
Li et al., 2014	MCF-7 and MDA-MB-231 breast cancer cells	*In vitro* study of drug effect on apoptosis	Lidocaine + cisplatin	Lidocaine enhanced cisplatin-induced apoptosis	3
Olsen et al., 2004	MCF-7	*In vitro* study of drug effect on proliferation	Phenytoin, phenobarbital, valproic acid, lamotrigine	Phenytoin, phenobarbital, and valproic acid inhibited proliferation, whereas lamotrigine did not	3
Papi et al., 2012	HT-29 and LoVo colon carcinoma cells	*In vitro* study of drug effect on proliferation, invasion, and apoptosis	Valproic acid + rexinoid IIF	Drug combination inhibited cell growth and invasion, induced apoptosis	3
Wedel et al., 2011	LNCaP; PC-3 prostate cancer cells	*In vitro* study of drug effect on cell behavior	Valproic acid + mTOR inhibitor RAD001	Valproic acid and RAD001 reduced cell adhesion and migration	3
Yoon et al., 2011	MCF10A, MCF10A-Bcl2, MDA-MB-436 breast epithelial, and cancer cells	*In vitro* study of drug effect on cell behavior	Tetracaine, lidocaine	Tetracaine and lidocaine inhibit microtentacle attachment, microfilament organization, and cell adhesion	3
Zhang et al., 2011	RM-1 prostate cancer cells	*In vitro* study of drug effect on E-cadherin-mediated cell migration	Valproic acid	Valproic acid promoted E-cadherin expression and inhibited cell migration.	3
Zhang et al., 2012	MDA-MB-231 breast cancer cells	*In vitro* study of drug effect on cell behavior	Valproic acid	Valproic acid inhibited cell migration with clinically relevant doses	3

**FIGURE 2:**
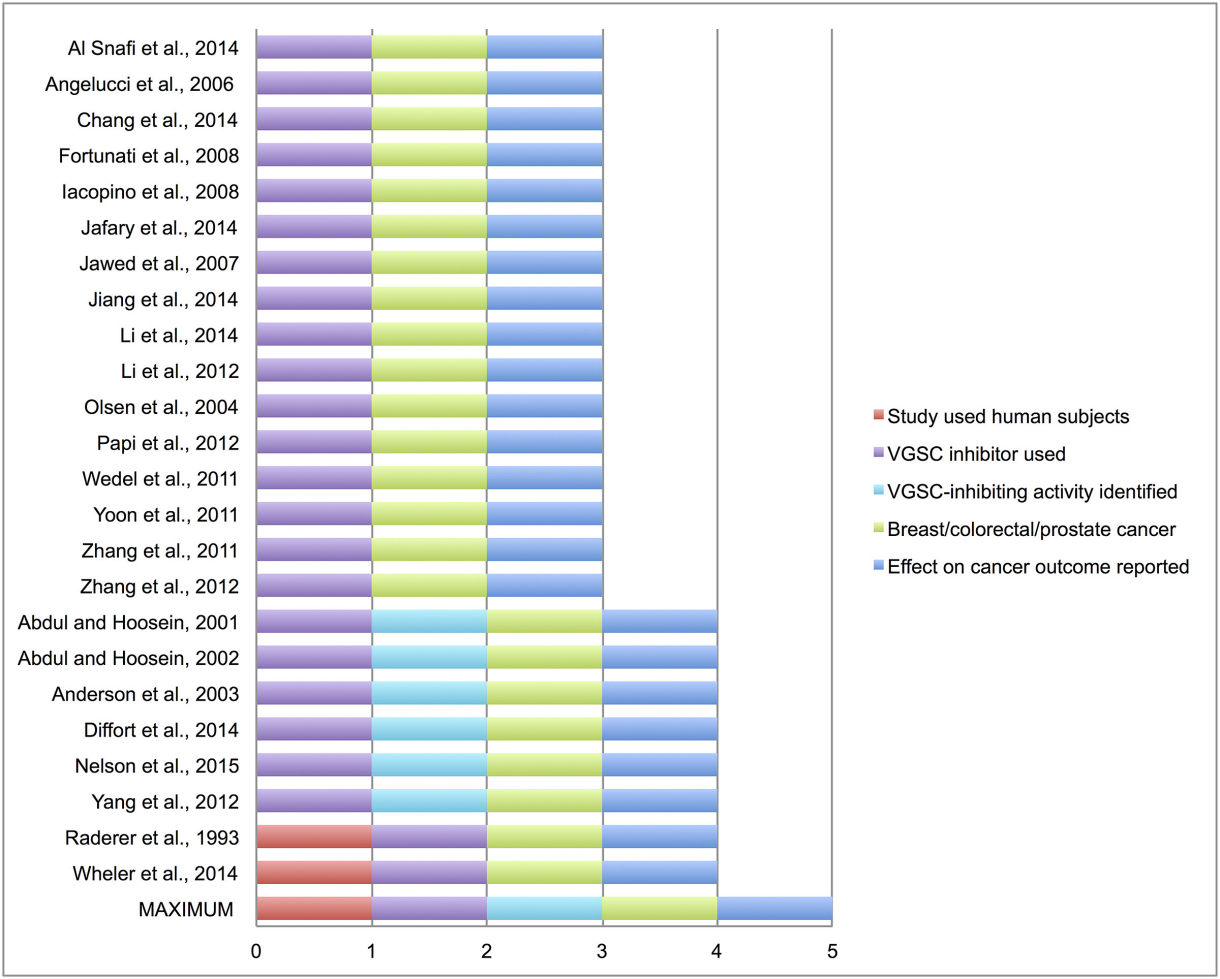
**Scoring of included studies**. Study quality and evidence was quantified in the included studies (2 clinical and 22 preclinical) according to the modified standardized pre-piloted criteria assessment tool in Supplementary Table S3. Maximum score = 5, minimum score for inclusion in systematic review = 3.

The remaining 22 papers scored between 3 and 4 out of five, and all of them were preclinical studies (**Figure 2**). Interestingly, four *in vitro* studies specifically tested the VGSC-inhibiting mode of action of the interventions (**Figure 2**). Three studies investigated prostate cancer cell lines and detected inhibited cell growth at clinically relevant drug doses of riluzole, sodium valproate, carbamazepine, phenytoin, and its derivatives (Abdul and Hoosein, 2001, 2002; Anderson et al., 2003), and one study used breast cancer cell lines and detected reduced migration and cell invasion with phenytoin (Yang et al., 2012). In the first study, Abdul and Hoosein (2001) showed that carbamazepine and phenytoin both reduced secretion of prostate-specific antigen (PSA) and interleukin-6 (IL-6) in prostate cancer cells. They found that valproate also inhibited PSA and IL-6 secretion to a lesser extent, although they attributed the effect of valproate to Ca^2+^ channel inhibition, rather than to its possible role as a VGSC inhibitor. All three drugs inhibited prostate cancer cell proliferation. In a subsequent study (Abdul and Hoosein, 2002), the same authors showed that riluzole also inhibited prostate cancer cell proliferation. However, in both studies, the authors did not directly show whether or not functional VGSCs were present in the tumor cells, e.g., by electrophysiological recording, nor did they provide evidence to indicate whether the drugs elicited their effects through VGSC inhibition or another, VGSC-independent mechanism. The third study (Anderson et al., 2003) showed that phenytoin and several other inhibitors (hydroxyamides and a hydantoin) reduced proliferation of an androgen-independent prostate cancer cell line, without significantly affecting viability. The authors also showed that the drugs inhibited Na^+^ current (i.e., VGSC functional activity) in *Xenopus* oocytes expressing Na_v_1.2, although they did not show whether the drugs inhibited endogenous VGSC activity in the prostate cancer cells. In the fourth study (Yang et al., 2012), we reported that phenytoin inhibited Na^+^ current, migration and invasion of metastatic MDA-MB-231 breast cancer cells at clinically relevant doses, although it had no effect on cell proliferation. Importantly, phenytoin had no effect on proliferation, migration or invasion of weakly metastatic MCF-7 cells, which do not express Na^+^ currents, suggesting that the effect of phenytoin is VGSC-dependent (Yang et al., 2012). All four studies suggest that further *in vivo* studies are warranted to explore the effect of VGSC-inhibiting drugs on cancer progression/metastasis.

There were two recent *in vivo* preclinical studies testing the effect of VGSC-inhibiting drugs on breast cancer metastasis in mice, which both scored 4 out of 5. In Nelson et al. (2015), we showed that phenytoin slowed tumor growth, local invasion and metastasis in an orthotopic mouse breast cancer model. In addition, Driffort et al. (2014) showed that ranolazine reduced lung colonization in an experimental metastasis mouse model of breast cancer. In the same study, ranolazine was also found to inhibit Na^+^ current, invasion and extracellular matrix degradation *in vitro*. Importantly, the effect of ranolazine on metastasis was equivalent to that of Na_v_1.5 down-regulation with shRNA, suggesting that the metastasis-inhibiting effect of this drug is VGSC-dependent.

**FIGURE 3:**
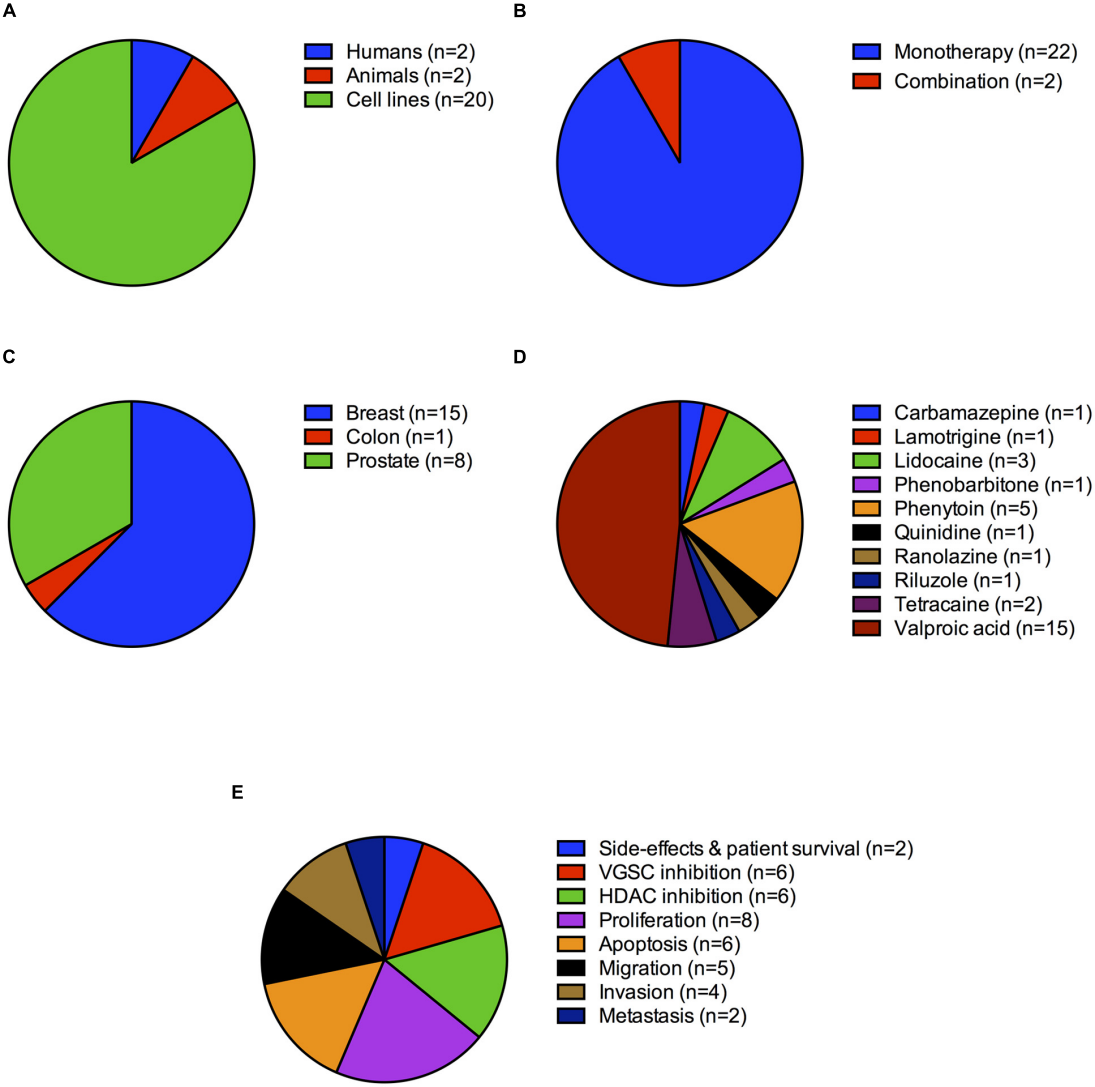
**Study distribution by cancer type, intervention and outcome measure. (A)** Number of studies (%) on humans, animals and cell lines. **(B)** Number of studies (%) testing monotherapy vs. those testing multiple drugs in combination. **(C)** Number of studies (%) on breast, colon and prostate cancer. **(D)** Studies (%) divided by drug type. **(E)** Studies (%) divided by outcome measure.

The remaining 17 studies scored 3 of 5 since they did not specifically look into the VGSC-inhibiting activity of the interventions. The most commonly tested drug was sodium valproate (13 studies) and the most common cell line breast cancer (11 studies). Not all drugs in these studies were tested at clinically relevant dosages, thus making the interpretation of some results and their clinical relevance challenging. For example, one study treated breast cancer cells with lidocaine in the range 0.01–1 mM (Li et al., 2014). However, lidocaine toxicity in humans has been reported at doses ≥0.04 mM (Collinsworth et al., 1974). Another study treated a range of tumor cell lines with sodium valproate at concentrations up to 10 times higher than the therapeutic range of 50–125 μg/ml, although growth inhibition was also observed at doses as low as 31 μg/ml for some, but not all cell lines tested (Al Snafi et al., 2014). Overall six studies reported pro-apoptotic effects, eight inhibition of cell proliferation, five inhibition of migration, and four inhibition of invasion. Four studies reported no effects on proliferation (**Figures 3A–E**).

## Discussion

It is known that VGSCs are expressed in metastatic cells in various tumor types (Brackenbury, 2012). In addition, a number of studies using the (non-therapeutic) VGSC-blocking neurotoxin tetrodotoxin (Grimes et al., 1995; Laniado et al., 1997; Fraser et al., 1999, 2003, 2004, 2005; Roger et al., 2003; Brackenbury and Djamgoz, 2006), gene knockdown, e.g., siRNA (Brackenbury et al., 2007; House et al., 2010; Brisson et al., 2013), or over-expression approaches (Bennett et al., 2004; Chioni et al., 2009), have generated considerable mechanistic insight into the role of VGSCs in metastatic cancer cells, reviewed in detail elsewhere (Fraser et al., 2014a; Besson et al., 2015). The purpose of this study was to systematically investigate current clinical evidence that VGSC-inhibiting drugs slow cancer progression, e.g., by inhibiting tumor growth and/or metastasis. Our initial aim was to focus on human studies, however, we soon discovered a scarcity of human data in this field. We therefore expanded our search to preclinical studies. To our knowledge this is the first systematic review in this field of interest.

Twenty-four studies met the inclusion criteria and attained high quality scores (≥3/5). Two were clinical drug trials, one of which was conducted in 1993 at a time when VGSC expression in cancer cells was not yet widely appreciated and therefore the VGSC-inhibiting mode of action of quinidine was not specifically investigated (Raderer et al., 1993). The second, more recent study investigated the HDAC inhibitory effects of sodium valproate as an adjuvant and its possible beneficial effect on survival (Wheler et al., 2014). Both studies included advanced disease, which would preclude the observation that VGSC inhibitors may elicit early anti-metastatic effects by slowing invasion and/or preventing cancer progression. Indeed, most systemic anti-metastatic therapies capitalize on the understanding of late stages of the metastatic cascade, once tumor cells have already spread to secondary sites (Mina and Sledge, 2011), so a VGSC-targeting therapy may be highly novel. Although the lack of clinical data was disappointing, the systematic review of preclinical data allowed us to expose the broad range of VGSC inhibitors that have now been tested in various models as potential anti-cancer drugs. We discovered that a variety of modes of action were tested/postulated, and there was a notable lack of standardization in the outcome measures that were captured.

A key theme of the preclinical *in vitro* studies was that various VGSC inhibitors generally had an inhibitory effect on proliferation of cancer cells, and/or promoted apoptosis, either alone, or in combination with other chemotherapeutic agents. In the majority of cases, the VGSC-inhibiting mode of action of these drugs was not tested/identified. Indeed, the mode of action of the most commonly studied VGSC-inhibiting drug, sodium valproate, was shown to be, at least partially, through HDAC inhibition in several studies (Angelucci et al., 2006; Zhang et al., 2011, 2012; Papi et al., 2012). Whilst this explanation is highly plausible, it cannot be excluded that valproate may also elicit its anti-tumor effects through additional mechanisms, including VGSC inhibition. In support of this notion, taking the general observation that different VGSC-inhibiting drugs, not all of which are HDAC inhibitors, have similar anti-cancer properties, their effects may be through another common mechanism, i.e., VGSC inhibition. A similar situation may occur for the Ca^2+^ channel blockers verapamil and diltiazem: these drugs elicit an anticancer effect by inhibiting P glycoprotein and multidrug resistance, but have also been shown to inhibit VGSCs in metastatic breast cancer cells (Roger et al., 2004). Further work is required to investigate these possibilities.

There were several key weaknesses with a number of the *in vitro* studies. Firstly, the methodology and outcome measures were not standardized across different studies, and interpretation of some measures was inconsistent, e.g., a relative reduction in cell number was variously interpreted as a reduction in proliferation, an increase in apoptosis, or an increase in cytotoxicity across different studies. Secondly, drug dosing was highly variable across different studies. Whilst some carefully reported the measured dose was within the therapeutic range for other indications (Yang et al., 2012; Nelson et al., 2015), others did not, and in several cases the dose was orders of magnitude above the therapeutic range (Al Snafi et al., 2014; Li et al., 2014), with the risk that the reported effects may be due to non-specific cytotoxicity. Clearly, further work is required to standardize dosing regimen across different *in vitro* models in order to unequivocally establish whether or not these drugs do indeed inhibit metastatic cell behaviors in different cancer cell types. Finally, the *in vitro* studies have generally focused on tumor cell lines in isolation without taking into consideration the possible effect of VGSC-inhibiting drugs on other cells in the heterogeneous tumor microenvironment, e.g., immune cells, or possible drug–drug interactions with concurrently administered chemotherapeutic agents (Vecht et al., 2003; Lo et al., 2012).

Despite the relatively large number of *in vitro* studies, our search revealed only two *in vivo* studies where VGSC inhibitors have been tested in mouse models (Driffort et al., 2014; Nelson et al., 2015). Both these studies employed xenografts of MDA-MB-231 breast cancer cells into immunocompromised mice, although one involved implantation at the orthotopic site and the other tail vein injection (spontaneous metastasis). Interestingly, both models showed a similar result, that the VGSC-inhibiting drugs tested inhibited metastatic dissemination of breast cancer cells. Similar to the *in vitro* studies, one weakness with these *in vivo* models is that they cannot consider the effects of the drugs on cell–cell interaction in a complex tumor microenvironment in immune-competent hosts (Vargo-Gogola and Rosen, 2007). Nonetheless, it is worthy to note here that one study has shown that the VGSC-inhibiting neurotoxin tetrodotoxin, not licensed for clinical use, so excluded from our review, inhibits lung metastasis in a rat prostate cancer allograft model, an immunocompetent host (Yildirim et al., 2012).

It is important to note that several studies have shown that compounds not classically considered as VGSC-inhibiting therapeutic agents, e.g., the natural plant phenolic resveratrol and the omega-3 long chain polyunsaturated docosahexaenoic acid, reduce VGSC-dependent cancer cell migration and invasion (Isbilen et al., 2006; Fraser et al., 2014b; Wannous et al., 2015). In addition, recent evidence suggests that anesthetics that target VGSCs, e.g., ropivacaine, may also inhibit cancer cell invasion (Baptista-Hon et al., 2014). Furthermore, the voltage-gated Ca^2+^ channel-targeting antiepileptic drug gabapentin, which may also inhibit VGSCs (Zhang et al., 2013), has recently been shown to inhibit invasion and metastasis of prostate cancer cells at high doses (Bugan et al., 2015). Finally, a recent study published in June 2015 (after the search period ended) has shown that lidocaine inhibits Na^+^ current, extracellular signal-regulated kinase (ERK) phosphorylation and cellular invasion of SW620 colorectal cancer cells *in vitro* (House et al., 2015). Thus, VGSC inhibition via the use of various agents beyond the scope of this review may also have therapeutic value, and further work is required to establish this possibility *in vivo*.

Based on this review, we make the following recommendations: Firstly, future clinical studies need to directly investigate the VGSC-inhibitory effects of relevant drugs using electrophysiological recording as an outcome measure. Second, clinically relevant *in vivo* models are needed to identify the most potent and safest VGSC-inhibiting drugs as anti-metastatic agents. Thirdly, methodology and outcome measures need to be standardized in order to be able to comparison of outcomes across tumor types and drugs. Finally, specific quality assessment tools are needed to evaluate *in vitro* studies of clinical interest.

## Conclusion

There is only one registered clinical trial in cancer patients specifically exploring the anti-tumor effects of licensed VGSC blockers on survival. The study in question is a randomized open-label trial in India exploring the effect of lidocaine administration during surgery on disease-free survival of patients with operable breast cancer and will not complete until 2019 (ClinicalTrials.gov Identifier: NCT01916317). Given the large and growing body of preclinical evidence in favor of VGSC inhibitors as anti-tumor and anti-metastatic agents, clinical trials are urgently needed to explore this novel therapeutic angle in breast, colorectal, and prostate cancer patients. Novel agents that preferentially target the neonatal splice variant of VGSCs expressed in these adult cancers (Fraser et al., 2005; Baptista-Hon et al., 2014) should be developed and assessed. In addition, a number of the VGSC inhibitors investigated here are already licensed for non-cancer and cancer indications and could repurposed in phase 2 trials specifically investigating their VGSC inhibitory and anti-cancer properties.

## Author Contributions

FM and IW had the original idea for this study. FM, CU, and WB conducted the analysis. FM, CU, and WB wrote the draft of the manuscript. IW and MB contributed to the development of the idea, and study design/interpretation. All authors approved the final submitted version of the manuscript.

## Conflict of Interest Statement

The authors declare that the research was conducted in the absence of any commercial or financial relationships that could be construed as a potential conflict of interest.
