# Supplementary material for: Therapeutic Value of Voltage-Gated Sodium Channel Inhibitors in Breast, Colorectal, and Prostate Cancer: A Systematic Review
Source: Front Pharmacol. 2015 Nov 12;6:273. doi: 10.3389/fphar.2015.00273 (PMC4714608; doi:10.3389/fphar.2015.00273)
Supplement: Supplementary file 2 [file Table_2.DOCX]

**Supplementary Table 2.** Search terms for Ovid MEDLINE In-Process & Other Non-Indexed Citations and Ovid MEDLINE 1946 to 2015 May 20.

| 1 | exp Colorectal Neoplasms/ |
| --- | --- |
| 2 | (colorectal and (neoplas* or cancer* or metasta* or tumor* or tumour* or carcino* or oncol*)).ti,ab. |
| 3 | ((colon or colonic) and (neoplas* or cancer* or metasta* or tumor* or tumour* or carcino* or oncol*)).ti,ab. |
| 4 | ((rectum or rectal) and (neoplas* or cancer* or metasta* or tumor* or tumour* or carcino* or oncol*)).ti,ab. |
| 5 | exp Prostatic Neoplasms/ |
| 6 | (prostat* and (neoplas* or cancer* or metasta* or tumor* or tumour* or carcino* or oncol*)).ti,ab. |
| 7 | exp Breast Neoplasms/ |
| 8 | ((breast or mamma*) and (neoplas* or cancer* or metasta* or tumor* or tumour* or carcino* or oncol*)).ti,ab. |
| 9 | 1 or 2 or 3 or 4 or 5 or 6 or 7 or 8 |
| 10 | exp Sodium Channel Blockers/ |
| 11 | ((sodium or NA) adj3 (blocker* or inhibitor*)).ti,ab,rn. |
| 12 | Phenytoin/ |
| 13 | Phenytoin or Dilantin or Diphenylhydantoin or Phenytoine or Aleviatin or Zentropil or Epamin or Diphantoin or Fenylepsin).ti,ab,rn. |
| 14 | Carbamazepine/ |
| 15 | (Carbamazepine or Tegretol or Carbamazepen or Finlepsin or Carbazepine or Tegretal or Biston or Epitol or Timonil).ti,ab,rn. |
| 16 | Aminoimidazole Carboxamide/ |
| 17 | (Colahepat or Carboxamide).ti,ab,rn. |
| 18 | (eslicarbazepine or Exalief or Zebinix).ti,ab,rn. |
| 19 | (oxcarbazepine or Trileptal or Oxcarbamazepine or Timox or Oxacarbazepine or Oxcarbazepina or Oxcarbazepinum or Oxcarbazepime or Epilexter).ti,ab,rn. |
| 20 | (Rufinamide or Inovelon or Banzel or Xilep or Banzel).ti,ab,rn. |
| 21 | Valproic Acid/ |
| 22 | ("Sodium valproate" or "Sodium 2-propylpentanoate" or "Valproate sodium" or "Valproic acid sodium salt" or Epilim or "Valproic acid sodium" or Eurekene).ti,ab,rn. |
| 23 | Lamotrigine or Lamictal or Lamotriginum or Labileno or Lamotrigina or Lamictal).ti,ab,rn. |
| 24 | (Lacosamide or Erlosamide or Harkoseride or Vimpat or Erlosamide).ti,ab,rn. (366) |
| 25 | (Topiramate or Topamax or Epitomax or Topiramatum or Tipiramate or Topimax or Topomax).ti,ab,rn. |
| 26 | Riluzole/ |
| 27 | (Riluzole or Rilutek or Riluzol or Riluzolum).ti,ab,rn. |
| 28 | (Ranolazine or Ranexa or Latixa or Ran4 or Ranexa).ti,ab,rn. |
| 29 | exp Anti-Arrhythmia Agents/ |
| 30 | ((Anti-Arrhythmia or Anti-Arrhythmic) and Agent*).ti,ab,rn. |
| 31 | Disopyramide/ |
| 32 | (Disopyramide or Dicorantil or Ritmodan or Rythmodan or Disopiramida or Disopyramidum or Isorythm or Lispine or Searle or Disopyramidum).ti,ab,rn. |
| 33 | Quinidine/ |
| 34 | (Chinidin or Pitayine or Kinidin or Conquinine or Coccinine or Conchinine or Quinidex or Quinidine).ti,ab,rn. |
| 35 | Procainamide/ |
| 36 | (Procainamide or Biocoryl or Novocainamide or Pronestyl or Novocamid or Procamide or Procaine amide or Novocainamid or Novocaine amide or Procan).ti,ab,rn. |
| 37 | Lidocaine/ |
| 38 | (Lidocaine or Lignocaine or Xylocaine or Lidoderm or Anestacon or Esracaine or L-Caine or Alphacaine or Cappicaine or Duncaine).ti,ab,rn. |
| 39 | Mexiletine/ |
| 40 | (Mexiletine or Mexiletina or Mexiletinum or Mexiletene or Mexityl or Mexiletinum or Mexiletina or 2-Propanamine).ti,ab,rn. |
| 41 | Tocainide/ |
| 42 | (Tocainide or Tocainida or Tocainidum or Tonocard or Alanyl-2,6-xylidide or Tocainidum or Tocainida or CHEBI:9611 or Taquidil).ti,ab,rn. |
| 43 | Moricizine/ |
| 44 | (Moricizine or Moracizine or Ethmozine or Moracizin or Moracizina or Moracizinum or EN-313 or Moracizinum or Moracizina).ti,ab,rn. |
| 45 | Flecainide/ |
| 46 | (Flecainide or Flecaine or Tambocor or Flecainida or Flecainidum or Flecainide or Flecainidum or Flecainida).ti,ab,rn. |
| 47 | Propafenone/ |
| 48 | (Propafenone or Propafenonum or Propafenona or Rythmol or Propafenonum or Propafenona).ti,ab,rn. |
| 49 | 10 or 11 or 12 or 13 or 14 or 15 or 16 or 17 or 18 or 19 or 20 or 21 or 22 or 23 or 24 or 25 or 26 or 27 or 28 or 29 or 30 or 31 or 32 or 33 or 34 or 35 or 36 or 37 or 38 or 39 or 40 or 41 or 42 or 43 or 44 or 45 or 46 or 47 or 48 |
| 50 | 9 and 49 |
