# Supplementary material for: Therapeutic Value of Voltage-Gated Sodium Channel Inhibitors in Breast, Colorectal, and Prostate Cancer: A Systematic Review
Source: Front Pharmacol. 2015 Nov 12;6:273. doi: 10.3389/fphar.2015.00273 (PMC4714608; doi:10.3389/fphar.2015.00273)
Supplement: Supplementary file 3 [file Table_3.DOCX]

**Supplementary Table 3.** Modified Quality Assessment Tool for Observational Cohort and Cross-Sectional Studies (mQATSO)

1. Did the study use human subjects?

| A. | No | 0 |
| --- | --- | --- |
| B. | Yes | 1 |

1. Did the study test the effect of at least one of the VGSC inhibitors listed in Table 1?

| A. | No | 0 |
| --- | --- | --- |
| B. | Yes | 1 |

1. Did the study include at least one of the index cancers (breast, colorectal and/or prostate)?

| A. | No | 0 |
| --- | --- | --- |
| B. | Yes | 1 |

1. Did the study measure the effect of the intervention on cancer survival or a metastasis-specific outcome, e.g. cellular proliferation, migration, invasion?

| A. | No | 0 |
| --- | --- | --- |
| B. | Yes | 1 |

1. Did the study specifically investigate the VGSC-inhibiting mode of action of the drug/intervention?

| A. | No | 0 |
| --- | --- | --- |
| B. | Yes | 1 |

Scoring method: Sum of scores in response to questions1-5.

Grading of the QATSO score:

| <3/5 | ≥3/5 |
| --- | --- |
| Excluded | Included |
